# Supplementary material for: Patterns of Intron Gain and Loss in Fungi
Source: PLoS Biol. 2004 Nov 30;2(12):e422. doi: 10.1371/journal.pbio.0020422 (PMC532390; doi:10.1371/journal.pbio.0020422)
Supplement: Table S1 — Also available at http://genes.mit.edu/NielsenEtAl/. (4.3 MB ZIP). [file pbio.0020422.st001.zip › NielsenEtAl/html/141.html]

AN4900.1.NCU00223.1.MG05306.1.FG06664.1


```
 CLUSTAL W (1.82) Multiple Sequence Alignments - Introns Inserted


Sequence 1: NCU00223.1	430 aa
Sequence 2: MG05306.1	410 aa
Sequence 3: FG06664.1	417 aa
Sequence 4: AN4900.1	382 aa
Alignment Length: 449 aa
Number Identitical Residues: 127 aa
Alignment Score (without introns) 5992


MG05306.1 	MSIDRKDPVKEDTPDT-VGSSP--EGEQQTQQGNSATNNGN------HQEPQETQQPKRK
NCU00223.1	MSIDKHDPTKDDSEDSQMESSP--EREREGEAPGGEANGPNTGTSTKTAAPQENQQPKRK
FG06664.1 	MSIDKADPAQHHNNNNHLNGLNGHPRDETDSLVGSSPEGDHHPAITSANATQEPQQPKRK
AN4900.1  	MAMDQVAPKSEPLNEGSISSAVS-TPDPEGE----------------VLTQDVAQTQKRK
          	*::*:  * ..   :  : .  .   :   .                    :  *  ***

MG05306.1 	GGRKP0IYATSEERKQRNRQAQAAFRERRTEYIKQLEEAIKIHEQNLHQLQAAHRNAADE
NCU00223.1	GGRKP0IYATSEERKQRNRQAQAAFRERRTEYIKQLEDAIRTHEQNLANLQAAHRHAADE
FG06664.1 	GGRKP0IYATSEERKQRNRQAQAAFRERRTEYIKQLEETIRVHESNLHNLQAAHRTAADE
AN4900.1  	GGRKP0IYATSEERKQRNRQAQAAFRERRTEYIRQLESTIKRNEESLQTLQQNHRTAADE
          	***** ***************************:***.:*: :*..*  **  ** ****

MG05306.1 	CLMLRYKNSLLERILLEKG1IDVQAELRAKTGSPNLGPTHMPQNLVPPPPISRSLLNRHH
NCU00223.1	CLMLRYKNSLLERILLEKG1IDVQAELQAKTGSPNLGPTHMPQNLVQQPPIQRALLNRHH
FG06664.1 	CLMLRYKNSLLERILLEKG~IDVQAELRAKTGSPNLGPTHMPQNIVQPPPIQRAIMNRHH
AN4900.1  	CLMLRYKNSLLERILLEKG1IDVQAELRLKAGTPN-GPGKPSPITTKAPSLQQAAISRSS
          	******************* *******: *:*:** ** : .   .  *.:.:: :.*  

MG05306.1 	-SRRSNSSIAPKMEPGLGV--LHP---------SPASRQGASSHSASPTNTNSIFTGSPA
NCU00223.1	-ARRSQSSIAPKLEPGIPISPLPPPVHSHASALSPKSRPTPLSHSASPSTTATFGSHSGA
FG06664.1 	QSRRSNSNIAPKAEP---VPVLPPPLQPHSNAASPKNRPTPSSHSNSPSNTGSAFSPAAS
AN4900.1  	-AQRHPSGLAPKEPFS-----VPQSRDGGFGIPSPQFQATPPSHVSSPSHAKSPNYGFQG
          	 ::*  *.:***   .     :  .     .  **  :  . **  **: : :      .

MG05306.1 	G-STSDHNQTNNRSGMPPGGMKPQQGVAMSQGLTPGGPARGMQQQMHTAAHRAHAMRVAA
NCU00223.1	SPVTSDGMIPPIRPHLPPASALKIHPPSQLGNGVPGLAQQQRQMQMPGLQQGQNHMRN--
FG06664.1 	DNMSMRGSMTSMSRQMPQQPHQPQQQ-----------QQQQQQMSQNTQAPRPSLMQN--
AN4900.1  	ALSPAGVDPQAQRSQMLTHSRNISQT------------SPPMSVGQPEPTEPKSAVSASM
          	   .           :        :                 .            :  : 

MG05306.1 	ATAGGATPSAPFYPSPAFQSHIDQL-----~-----E1QEYDAADNMMDD-CETPDTPSG
NCU00223.1	-SVGGNTNS--FYPTPSFQNHIEQLGKLTR1SFRNSE~QEYDAAAEMMDDPGETPDTPSG
FG06664.1 	SSRGQAGSGASYYPTPAFQNHIEQL-----~-----E1QEYDAQADMIDE--PEIETPAG
AN4900.1  	GSRAPRLPS-AYYPSP-FQKHYDQL-----~-----E1QEYDAQADMIDD---EHESSVG
          	.: .    . .:**:* **.* :**           * *****  :*:*:     ::. *

MG05306.1 	PGPYPGAPYSGEGQPLNLSSPAATGT--PLDSSIAPSPAVTSTATHTTSAYPSMTQLLDT
NCU00223.1	PGPYPGQAYGGDPQAMSLSSPVTSG---PQGNQQSP---IENGSHQQHPAYPPMAQLLDH
FG06664.1 	HGGYP-SGYSGENQQPMMMSPASSGPGHQMTPSHEPVQQQQQQPQTSHSQYPSMTQLLDQ
AN4900.1  	TSSFV-PGYNPSSSVSNASHPMNPHG--MNPYNHSSGEAVNGAYGNTSAMMGNYEPMLDA
          	 . :    *. . .      *  .        .  .            .       :** 

MG05306.1 	-GIDW-DPFGLTASMAFPNGS~QFAFDTSNMR--
NCU00223.1	-QYDFGDPFGLSASMAFP--T~QFSFDTSNMR--
FG06664.1 	NGLDW-DPFGLSASMAFPNGQ~QFQFEQANMR--
AN4900.1  	------DPFGLSA-------K1QFILVHGASPRL
          	      *****:*         ** :  .
```
